# Efeitos do Ato de Fumar na Mortalidade de Longo Prazo após Infarto do Miocárdio por Elevação de ST

**DOI:** 10.36660/abc.20201036

**Published:** 2021-11-17

**Authors:** Emrullah Kızıltunç, Yusuf Bozkurt Şahin, Salih Topal, Mehmet Akif Düzenli, Ekrem Karakaya, Nazif Aygül, Ramazan Topsakal, Kurtuluş Özdemir, Adnan Abacı

**Affiliations:** 1 Gazi University Faculdade de Medicina Departamento de Cardiologia Ancara Turquia Gazi University , Faculdade de Medicina , Departamento de Cardiologia , Ancara - Turquia; 2 Necmettin Erbakan University Faculdade de Medicina Departamento de Cardiologia Cônia Turquia Necmettin Erbakan University , Faculdade de Medicina , Departamento de Cardiologia , Cônia - Turquia; 3 Karabük University Faculdade de Medicina Departamento de Cardiologia Karabük Turquia Karabük University , Faculdade de Medicina , Departamento de Cardiologia , Karabük - Turquia; 4 Selçuk University Faculdade de Medicina Departamento de Cardiologia Cônia Turquia Selçuk University , Faculdade de Medicina , Departamento de Cardiologia , Cônia - Turquia; 5 Erciyes University Faculdade de Medicina Departamento de Cardiologia Caiseri Turquia Erciyes University , Faculdade de Medicina , Departamento de Cardiologia , Caiseri - Turquia; 6 KTU Karatay University Medicana Hospital Departamento de Cardiologia Cônia Turquia KTU Karatay University , Medicana Hospital , Departamento de Cardiologia , Cônia - Turquia

**Keywords:** Tabagismo, Nicotina/efeitos adversos, Infarto do Miocárdio com Supradesnivel do Segmento ST/complicações, Fatores de Risco, Mortalidade

## Abstract

**Fundamento:**

O paradoxo do fumante tem sido motivo de debate para pacientes com infarto agudo do miocárdio (IM) há mais de duas décadas. Embora haja muitas evidências demonstrando que não existe tal paradoxo, publicações defendendo desfechos melhores em fumantes pós-IM ainda são lançadas.

**Objetivo:**

Explorar o efeito do fumo na mortalidade de longo prazo após infarto do miocárdio por elevação de ST (STEMI).

**Métodos:**

Este estudo incluiu pacientes com STEMI que foram diagnosticados entre 2004 e 2006 em três centros terciários. Os pacientes foram categorizados de acordo com a exposição ao tabaco (Grupo 1: não-fumantes; Grupo 2: <20 pacotes*anos; Grupo 3: 2-040 pacotes*anos; Grupo 4: >40 pacotes*anos). Um modelo de regressão de Cox foi utilizado para estimar os riscos relativos para mortalidade de longo prazo. O valor de p <0,05 foi considerado como estatisticamente significativo.

**Resultados:**

Trezentos e treze pacientes (201 fumantes e 112 não-fumantes) foram acompanhados por um período médio de 174 meses. Os fumantes eram mais novos (54±9 vs. 62±11, p: <0,001), e a presença de fatores de risco cardiometabólicos foi mais prevalente entre os não-fumantes. Uma análise univariada do impacto do hábito de fumar na mortalidade revelou uma curva de sobrevivência melhor no Grupo 2 do que no Grupo 1. Porém, após ajustes para fatores de confusão, observou-se que os fumantes tinham um risco de morte significativamente maior. O risco relativo tornou-se maior de acordo com a maior exposição (Grupo 2 vs. Grupo 1: RR: 1,141; IC95%: 0,599 a 2.171; Grupo 3 vs. Grupo 1: RR: 2,130; IC95%: 1,236 a 3,670; Grupo 4 vs. Grupo 1: RR: 2,602; IC95%: 1,461 a 4,634).

**Conclusão:**

O hábito de fumar gradualmente aumenta o risco de mortalidade por todas as causas após STEMI.

## Introdução

A associação causal entre o ato de fumar e doenças cardiovasculares ateroscleróticas, malignidades e doenças pulmonares parenquimatosas já foi documentada.^1^ Apesar desta clara associação, o paradoxo do fumante (desfechos melhores após alguma doença potencialmente fatal entre fumantes *versus* não-fumantes) tem sido tópico de debates há muitos anos. Apesar da crescente evidência demonstrando que não existe um real paradoxo do fumante, há publicações recentes alegando que existe, sim, um paradoxo do fumante para alguns tipos de doenças ou grupos de pacientes, como aqueles com infarto agudo do miocárdio, derrame, além de indivíduos que se submeteram a um implante de transcateter da válvula aórtica (TAVI).^2 - 4^ A baixa reatividade de plaquetas, a atividade antiplaquetária melhorada com uso do clopidogrel e a possível ativação de vias pré-condicionantes com a hipóxia induzida pelo fumo estão sendo sugeridas como base do paradoxo do fumante.^5 , 6^ Além disso, sugere-se que algumas disparidades regionais possam ser encontradas no paradoxo do fumante.^7 , 8^

Neste estudo, nosso objetivo foi explorar o efeito do cigarro com uma abordagem dose-dependente na mortalidade de longo prazo entre sobreviventes de um primeiro STEMI.

## Métodos

### Pacientes

Este estudo retrospectivo incluiu os primeiros pacientes com STEMI acompanhados em três hospitais universitários na Turquia entre 2004 e 2006 (Gazi University, Faculdade de Medicina, Departamento de Cardiologia, Ancara; Erciyes University, Faculdade de Medicina, Departamento de Cardiologia, Caiseri; Selçuk University, Faculdade de Medicina, Departamento de Cardiologia, Cônia). O estudo foi conduzido de acordo com a Declaração de Helsinki, e a aprovação foi obtida pelo Comitê de Ética da Gazi University, Faculdade de Medicina, além de outros departamentos participantes. O STEMI foi definido como: presença de dor típica no peito ou sintoma equivalente; presença de elevação de ST ≥ 2 mm em pelo menos duas derivações contínuas e/ou maiores concentrações de CK-MB. Pacientes que tinham indicação para reperfusão imediata foram tratados com terapia trombolítica ou intervenção coronariana percutânea (ICP) primária. Uma ICP de resgate foi realizada em pacientes nos quais a terapia trombolítica não foi bem-sucedida. Aqueles que apresentaram alívio nos sintomas e cujo ECG estava consistente com STEMI subagudo na apresentação, assim como pacientes cuja trombólise foi exitosa, foram submetidos à angiografia coronária após 24 horas da admissão hospitalar. Todos os pacientes receberam ácido acetilsalicílico 100 – 300 mg, clopidogrel 75 mg e estatina (atorvastatina, sinvastatina, pravastatina ou fluvastatina) na alta. Mais de 90% dos pacientes usavam betabloqueadores (metoprolol ou carvedilol) e tratamento com bloqueadores de renina e angiotensina na alta, a menos que houvesse alguma contraindicação ou intolerância a esses agentes.

Durante o período do estudo, todos os pacientes com STEMI foram recrutados consecutivamente para uma base de dados. Todos os pacientes foram questionados sobre sintomas, principais fatores de risco, doença coronariana e histórico médico. Os achados dos exames físicos e laboratoriais, incluindo hemograma, níveis de creatinina, níveis de glicose, lipídeos séricos, picos de creatina-quinase MB foram registrados na primeira internação. Dados ecocardiográficos e angiográficos foram coletados. Para informações sobre o fumo, pacientes foram questionados se já haviam fumado um cigarro; se sim, o número de cigarros por dia e a duração do ato de fumar foram registrados, e, assim, a dose de exposição ao fumo foi calculada como pacotes*anos [número de cigarros por dia/20)*anos de uso do cigarro]. Os pacientes foram categorizados de acordo com a quantidade de exposição ao cigarro (Grupo 1: fumantes recentes; Grupo 2: <20 pacotes*anos; Grupo 3: 20-40 pacotes*anos; Grupo 4: >40 pacotes*anos). Os seguintes pacientes foram excluídos da análise de sobrevida: aqueles diagnosticados com infarto do miocárdio por elevação não-ST; pacientes com alguma doença cardíaca diferente do STEMI (insuficiência cardíaca, doença valvular cardíaca); aqueles com histórico de infarto do miocárdio ou procedimento prévio de revascularização coronária; e pacientes com imagem prejudicada no ecocardiograma transtorácico. Indivíduos cuja informação sobre o fumo não estava no prontuário de hospitalização inicial (incluindo cigarros consumidos por dia e anos de fumo), aqueles sem informação de sobrevida e os que morreram na primeira internação foram excluídos do estudo.

### Ecocardiograma

Os ecocardiogramas foram realizados com média de 2 dias (percentis 25 e 75 de 1 a 3 dias) após a admissão utilizando o sistema Vingmed CFM System Five (GE Medical. Horten, Noruega), com um transdutor 2,5 MHz ou modelo 5000, Advanced Technology Laboratories Inc; (Bothell, WA), com um transdutor de 2 a 4 MHz, sendo gravado em mídia digital. Paraesternal eixo longo e curto, assim como cortes apicais 2 e 4 câmaras, foram registrados na posição lateral esquerda em repouso. O ventrículo esquerdo foi avaliado de acordo com o modelo de 16 segmentos, de acordo com proposta da Sociedade Americana de Ecocardiografia.^9^ A motilidade regional da parede em cada segmento foi visualmente avaliada, utilizando um sistema de pontuação de quatro pontos: 1= normal, motilidade de parede normal; 2= hipocinesia, redução marcada da motilidade da parede endocardial; 3= acinesia, ausença de motilidade da parede interna; 4= discinesia, motilidade de parede paradoxal contrária ao lúmen ventricular esquerdo em sístole. Se mais de dois segmentos na zona de infarto, ou 4 ou mais em todos os 16 segmentos, não fossem visualizados, o estudo era considerado inadequado e esses pacientes não eram incluídos. O índice de motilidade da parede (WMSI) ventricular esquerda e a fração de ejeção ventricular esquerda (FEVE) foram utilizados para avaliar a extensão da disfunção sistólica no ventrículo esquerdo. O WMSI foi calculado ao dividir a soma da pontuação dos segmentos pelo número de segmentos visualizados. O método de Simpson modificado foi usado para medir a FEVE. A disfunção ventricular esquerda grave foi definida quando a FEVE <40%. Todos os ecocardiogramas foram analisados por dois observadores experientes que não tiveram acesso aos dados clínicos e angiográficos.

### Angiografia coronária

A angiografia coronária foi realizada pelo acesso femoral utilizando a técnica padrão de Judkins. A estenose na artéria coronariana foi visualmente estimada por dois observadores independentes sem acesso à identidade e às informações clínicas dos pacientes. A localização da lesão culpada foi determinada pela angiografia coronária. A doença em um único vaso foi definida pelo diâmetro da estenose maior que 50% em somente uma artéria coronária. A doença de dois e três vasos foi definida de acordo com os mesmos critérios. A doença principal esquerda foi considerada como a doença de dois vasos.

### Informação de sobrevida

Em maio de 2020, os dados de sobrevida dos pacientes foram coletados pelo sistema eletrônico de notificação de mortes, de forma retrospectiva. Em nosso país, todas as mortes devem ser registradas no sistema eletrônico do governo com um número de identificação pessoal. Assim, o sistema oferece dados robustos sobre as informações de sobrevivência e data de morte. A duração do acompanhamento foi calculada ao subtrair a data do diagnóstico da data de morte nos pacientes falecidos, e ao subtrair a data do diagnóstico do dia 1º de maio, 2020, nos pacientes vivos.

### Análise estatística

O software SPSS 22.0 para Windows foi usado para análise dos dados. Para as variáveis contínuas, a normalidade da distribuição foi testada utilizando o teste de Kolmogorov-Smirnov. Os resultados foram apresentados como média ± desvio padrão (DP) para variáveis com distribuição normal, e como mediana (intervalo interquartil 25-75) para variáveis com distribuição anormal. As variáveis categóricas foram apresentadas em números e porcentagem. Para a comparação das variáveis contínuas entre fumantes e não-fumantes, as amostras independentes do teste t ou do teste U de Mann-Whitney foram utilizadas quando apropriado. As variáveis categóricas foram analisadas usando o teste de qui-quadrado ou o teste exato de Fisher. O teste de log-rank foi usado para detectar os efeitos univariados das variáveis sobre mortalidade específicas do estudo. As estimativas de sobrevida de Kaplan-Meier foram calculadas. Os possíveis fatores identificados nas análises univariadas foram, depois, incluídas na análise de regressão de Cox para determinar os preditores independentes da mortalidade por todas as causas. O valor de p <0,05 foi considerado como estatisticamente significativo.

## Resultados

Trezentos e treze pacientes consecutivos diagnosticados com STEMI agudo ou subagudo foram incluídos no estudo. A duração mediana do acompanhamento foi de 14,5 anos. Cento e doze (35,8%) pacientes nunca tinham fumado antes da internação inicial (Grupo 1). A quantidade da exposição dos fumantes na internação inicial foi de: 66 pacientes (21,1%) <20 pacotes*anos (Grupo 2); 94 pacientes (30,0%) 20-40 pacotes*anos (Grupo 3); 41 pacientes (13,1) >40 pacotes*anos (Grupo 4).

Aspectos demográficos de base e parâmetros laboratoriais dos fumantes e não-fumantes estão demonstrados na Tabela 1 . Os fumantes eram mais novos e, mais frequentemente, do sexo masculino. Hipertensão e diabetes foram mais prevalentes entre os não-fumantes, e o histórico familiar para a doença arterial coronariana prevaleceu entre os fumantes. Enquanto os níveis de hemoglobina no momento da internação estiveram mais altos entre os fumantes em comparação aos não-fumantes, os níveis de colesterol total, LDL e glicose estavam mais altos no momento da internação em não-fumantes do que em fumantes. O local do infarto, receber uma reperfusão imediata, níveis de pico de creatina-quinase e CKMB, escore da área relacionada ao infarto e escore de Gensini foram similares entre fumantes e não-fumantes ( Tabela 2 ). A fração de ejeção (FE) média foi maior entre fumantes.

**Tabela 1:** – Aspectos demográficos e laboratoriais de base para fumantes e não-fumantes

Variáveis	Não-fumantes (112)	Fumantes (201)	p
Idade (Anos)	62±11	54±9	<0,001
Sexo (Feminino)	30(26,8)	16(8,0)	<0,001
Hipertensão	52(46,4)	46(22,9)	<0,001
Diabetes	28(25,0)	18(9,0)	<0,001
Histórico familiar de DAC	17(15,2)	50(24,9)	0,045
Exposição ao tabaco - Nível			
<20 pacotes-anos	-	66(32,8)	
20-40 pacotes-anos	-	94(46,8)	
>40 pacotes-anos	-	41(20,4)	
Hemoglobina, g/dl	14,0±1,7	14,7±1,5	<0,001
CGB*10^3^	10,8±3,3	11,8±4,0	0,022
Creatinina, mg/dl	1,08±0,26	1,03±0,22	0,120
Colesterol total, mg/dl	198±44	187±41	0,025
LDL, mg/dl	131±37	121±36	0,017
HDL, mg/dl	41±10	40±11	0,521
Triglicérides, mg/dl	111(68-161)	113(82-164)	0,296
Glicose na admissão hospitalar, mg/dl	148±74	125±45	0,002

*DAC: doença arterial coronariana; HDL: lipoproteína de alta densidade; LDL: lipoproteína de baixa densidade; CGB: contagem de glóbulos brancos. Variáveis contínuas foram apresentadas como média±DP ou mediana (IIQ 25-75); variáveis categóricas foram apresentadas em números (%).*

**Tabela 2:** – Aspectos clínicos, angiográficos e ecocardiográficos em fumantes e não-fumantes

Variáveis	Não-fumantes (112)	Fumantes (201)	p
IM prévio, n(%)	65(58,0)	112(55,7)	0,692
Trombólise + ICP primária, n(%)	90(80,4)	154(76,6)	0,444
Pico de CK, U/l	2065(1239-2955)	2170(1361-3396)	0,253
Pico de CK-MB, U/l	189(122-286)	225(134-360)	0,149
Fração de Ejeção,%	47±10	50±9	0,036
Índice de motilidade da parede	1,59±0,36	1,57±0,34	0,584
IRA, n(%)			
ADA	65(58,0)	113(56,2)	0,782
AC	8(7,1)	19(9,5)	
ACD	39(34,8)	69(34,3)	
Gensini	39(24-55)	38(18-52)	0,213
FE <40%, n(%)	21(18,8)	31(15,4)	0,448
Morte, n(%)	38(33,9)	70(34,8)	0,873

*CK: creatina-quinase; AC: artéria circunflexa; FE: fração de ejeção; IRA: artéria relacionada ao infarto; ADA: artéria descendente anterior esquerda; IM: infarto do miocárdio; ACD: artéria coronária direita. Variáveis contínuas foram apresentadas como média±DP ou mediana (IIQ 25-75); variáveis categóricas foram apresentadas em números (%).*

**Figura 1:**
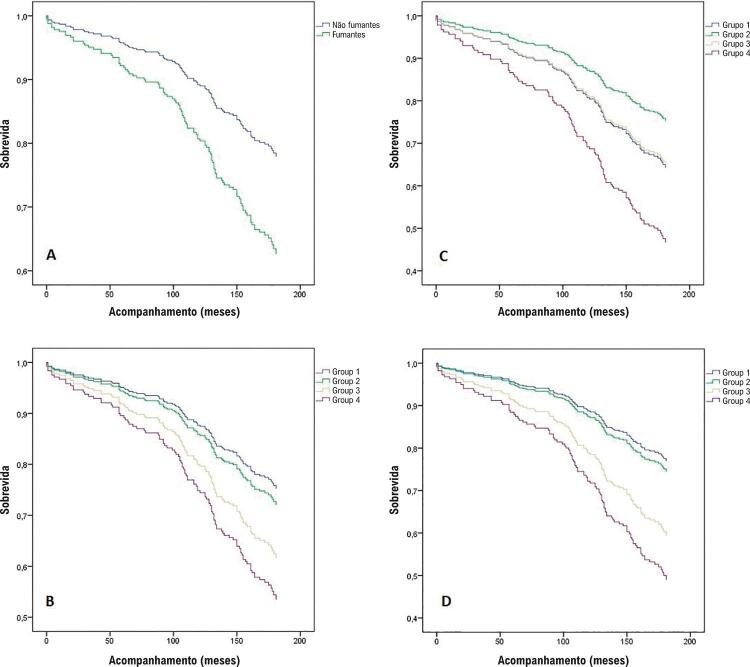
– Curvas de sobrevivência de Kaplan-Meier em fumantes e não-fumantes: Fumantes e não-fumantes geral (A); curvas não-adjustadas para grupos de fumantes (B); ajustado por idade (C); ajustado por idade, hipertensão, diabetes, hemoglobina, artéria relacionada a infarto, reperfusão imediata e presença de FE do ventrículo esquerdo reduzida (D); Grupo 1: não-fumantes, Grupo 2: <20 pacotes*anos, Grupo 3: 20-40 pacotes*anos, Grupo 4: >40 pacotes*anos.

Durante o acompanhamento, a morte ocorreu em 108 (34,5%) pacientes; 38 (33,9%) mortes entre não-fumantes; 70 (34,8%) mortes ocorreram entre os fumantes (p=0,873). A Tabela 3 mostra as variáveis do estudo em pacientes vivos e falecidos. No grupo dos falecidos, os pacientes eram mais velhos, tinham níveis de hemoglobina mais baixo na internação, FE mais baixa e WMSI mais alto após o evento isquêmico. Ser do sexo feminino, fumante compulsivo e apresentar FE <40% também foram fatores mais prevalentes no grupo dos falecidos. A Tabela 4 demonstra a análise de regressão multivariada de Cox para mortalidade por todas as causas. A idade avançada, o hábito de fumar e ter FE <40% após o infarto foram os preditores independentes da mortalidade de longo prazo. O risco relativo da mortalidade aumentou na análise dose-dependente em fumantes em comparação aos não-fumantes (Grupo 2 vs. Grupo 1: RR 1,141; IC95%: 0,599 a 2,171; Grupo 3 vs. Grupo 1: RR: 2,130; IC95%: 1,236 a 3,670; Grupo 4 vs. Grupo 1; RR: 2,602; IC95%: 1,461 a 4,634). As curvas de sobrevida de fumantes e não-fumantes são demonstrados na Figura 1 . Na Figura 1A, observamos as curvas de sobrevida de fumantes e não-fumantes. Na Figura 1B, observamos a análise não-ajustada, que demonstrou que o Grupo 2 tinha uma curva de sobrevivência melhor que o Grupo 1. Porém, após o ajuste por idade (Figura 1C) e todos os outros fatores de confusão (idade, hipertensão, diabetes, hemoglobina, artéria relacionada ao infarto, local do infarto, receber uma reperfusão imediata e presença de FE do ventrículo esquerdo) (Figura 1D), as curvas de sobrevida demonstraram o risco dose-dependente aumentado do ato de fumar.

**Tabela 3:** – Aspectos clínicos, demográficos, angiográficos e ecocardiográficos de pacientes vivos e falecidos

Variável (número)	Vivos (205)	falecidos (108)	p
Idade, anos	54±10	62±10	<0,001
Sexo (Feminino)	23(11,2)	23(21,3)	0,017
Hipertensão	55(26,8)	43(39,8)	0,019
Diabetes	28(13,7)	18(16,7)	0,475
Histórico familiar de DAC	45(22,0)	22(20,4)	0,746
Exposição ao tabaco - nível			0,021
Não-fumante	74(36,1)	38(35,2)	
<20 pacotes-anos	50(24,4)	16(14,8)	
20-40 pacotes-anos	62(30,2)	32(29,6)	
>40 pacotes-anos	19(9,3)	22(20,4)	
Hemoglobina, g/dl	14,6±1,5	14,2±1,8	0,027
CGB*10^3^	11,4±3,6	11,7±4,2	0,471
Creatinina, mg/dl	1.04±0.21	1,07±0,27	0,216
Colesterol total, mg/dl	194±44	185±40	0,096
LDL, mg/dl	126±37	121±36	0,336
HDL, mg/dl	41±10	41±13	0,683
Triglicérides, mg/dl	115(82-175)	106(73-146)	0,055
Glicose na admissão, mg/dl	132±63	137±49	0,566
IM prévia	118(57,6)	59(54,6)	0,619
Trombólise + ICP primária	163(79,5)	81(75)	0,360
Pico de CK, U/l	2156(1308-2999)	2172(1368-3726)	0,269
Pico de CK-MB, U/l	202(118-300)	232(141-379)	0,050
Fração de ejeção, %	50±9	47±10	0,016
Índice de motilidade da parede	1,54±0,31	1,64±0,40	0,013
IRA			0,979
ADA	117(57,1)	61(56,5)	
AC	18(8,8)	9(8,3)	
ACD	70(34,1)	38(35,2)	
Score Gensini	38(19-52)	38(21-57)	0,396
FE <40%	25(12,2)	27(25,0)	0,004

*DAC: doença arterial coronariana; CK: creatina-quinase; AC: artéria circunflexa; FE: fração de ejeção; HDL: lipoproteína de alta densidade; IRA: artéria relacionada ao infarto; ADA: artéria descendente anterior esquerda; LDL: lipoproteína de baixa densidade; IM: infarto do miocárdio; ACD: artéria coronária direita; CGB: contagem de glóbulos brancos. Variáveis contínuas foram apresentadas como média±DP ou mediana (IIQ 25-75); variáveis categóricas foram apresentadas em números (%).*

**Tabela 4:** – Análise de regressão multivariada de Cox para mortalidade de longo prazo

	Exp(B)	IC95% por Exp(B)		p
		Menor	Maior	
Idade	1,063	1,040	1,088	0,000
Sexo (Feminino)	1,730	0,985	3,040	0,056
Diabetes Mellitus	1,264	0,740	2,158	0,391
Hipertensão	1,184	0,765	1,832	0,448
Fumo				0,003
Grupo 2 vs. Grupo 1	1,141	0,599	2,171	0,689
Grupo 3 vs. Grupo 1	2,130	1,236	3,670	0,006
Grupo 4 vs. Grupo 1	2,602	1,461	4,634	0,001
Hemoglobina	0,978	0,856	1,118	0,749
Parede do Infarto (não-anterior vs. anterior)	0,771	0,257	2,307	0,641
Reperfusão imediata (apresentação aguda vs. subaguda)	0,978	0,622	1,538	0,924
IRA				0,825
ACD vs. ADA	1,305	0,428	3,982	0,640
AC vs. ADA	1,089	0,313	3,790	0,894
FE<40%	1,967	1,216	3,181	0,006

AC: artéria circunflexa; FE: fração de ejeção; IRA: artéria relacionada ao infarto; ADA: artéria descendente anterior esquerda; ACD: artéria coronária direita. Grupo 1: não-fumantes, Grupo 2: <20 pacotes*anos, Grupo 3: 20-40 pacotes*anos, Grupo 4: >40 pacotes*anos.

## Discussão

O principal achado deste estudo é o de que, dentre pacientes que sobreviveram após o STEMI, aqueles que fumavam antes do infarto apresentaram maior mortalidade por todas as causas do que os que nunca fumaram. O risco relativo de mortalidade aumenta conforme cresce a exposição ao cigarro. Outros preditores independentes são idade e presença de função sistólica do ventrículo esquerdo reduzida após o IM.

Muitos estudos epidemiológicos demonstraram que o risco associado ao ato de fumar aumenta de forma dose-dependente.^10 , 11^ Apesar disso, sugeriu-se que fumantes têm melhor prognóstico em algumas situações clínicas. Além dos pacientes com IM agudo, a presença do paradoxo do fumante tem sido definida em pacientes com derrame isquêmico, indivíduos com parada cardíaca com ressuscitação e pacientes com TAVI.^12 - 14^ A presença do paradoxo do fumante em pacientes com IM agudo é controversa. Os primeiros estudos sobre o paradoxo do fumante foram publicados na era trombolítica. Alguns desses estudos sugeriram que o ato de fumar era independentemente associado de desfechos favoráveis, e outros revelaram que os fumantes tinham desfechos favoráveis somente em análises univariadas.^15 , 16^ A estrutura da lesão culpada em fumantes (maior carga trombótica em fumantes do que em não-fumantes) foi discutida como uma potencial explicação para o paradoxo do fumante em pacientes com IM agudo. Sugeriu-se que os trombos em lesões culpadas entre os fumantes têm a tendência à lise espontânea e/ou à resposta na terapia trombolítica, o que seria melhor entre fumantes do que não-fumantes.^17^

Os resultados dos estudos durante a ICP primária também foram controversos. Alguns estudos demonstraram que fumantes e não-fumantes tinham taxas de mortalidade semelhantes; outros sugeriram que o ato de fumar esteve associado a desfechos favoráveis.^18 , 19^ Além disso, alguns estudos mostraram que fumantes apresentaram desfechos piores.^20^ Por exemplo, demonstrou-se que fumar era um preditor independente da mortalidade hospitalar mais baixa em pacientes com IM agudo, inclusive depois da análise múltipla para controlar potenciais fatores de confusão.^21^ Outra análise descobriu que fumantes apresentam resposta inflamatória aguda mais baixa, melhor reperfusão microvascular e taxas de mortalidade de 30 dias melhores no cenário do IM agudo.^3^ A resposta ao clopidogrel em fumantes tem sido o mecanismo mais popular para o paradoxo do fumante na era ICP primária. A reatividade mais baixa das plaquetas foi observada em fumantes tratados com clopidogrel em comparação a não fumantes tratados com clopidogrel.^22 , 23^ Sugeriu-se que as disparidades regionais para o paradoxo do fumante foram apresentadas, e esta hipótese se baseou na possível variabilidade genética do metabolismo do clopidogrel em diferentes raças.^8^

Embora haja muitos estudos abordando o paradoxo do fumante, há análises bem elaboradas que demonstram achados opostos. Resultados de cinco anos do ensaio SYNTAX demonstrou que o ato de fumar esteve associado a desfechos negativos após a revascularização em pacientes com doença arterial coronariana complexa.^24^ O ensaio ACUITY estabeleceu que, dentre os pacientes diagnosticados com infarto do miocárdio por elevação não-ST, aqueles que fumavam apresentavam uma taxa de mortalidade em um ano maior do que os que não fumavam.^25^ A taxa de mortalidade entre fumantes foi significativamente maior do que em não-fumantes em um grande registro de pacientes com STEMI que foram atendidos com ICP primária.^26^ Além disso, estudos de ressonância magnética do coração revelaram que, dentre pacientes com STEMI, aqueles que fumavam tinham taxas mais altas de hemorragia do miocárdio e piores desfechos cardiovasculares.^27^ Estudos recentes rejeitaram a presença do paradoxo do fumante em pacientes com insuficiência cardíaca e em pacientes com derrame isquêmico agudo.^28 , 29^ Finalmente, o resultado do estudo publicado por Kim et al.,^30^ foi importante. Eles descobriram que a diferença da resposta ao clopidogrel teve muita relação com a diferença nos níveis de hemoglobina. Encontraram reatividade de plaquetas semelhante entre fumantes e não-fumantes após ajustar a influência da hemoglobina na reatividade das plaquetas. ^30^

Em nosso estudo, a análise não-ajustada revelou que fumantes leves tinham melhor curva de sobrevida do que não-fumantes. Isso pode levar à ideia de que fumar pouco pode ser um bom hábito, mas, após ajustes de acordo com fatores de confusão, o ato de fumar aumentou o risco de mortalidade de forma dose-dependente. Então, fatores de confusão de base são muito importantes para o paradoxo do fumante. Como mencionamos, muitos dos estudos revelaram que os desfechos favoráveis atribuídos ao fumo desapareceram após a análise multivariada. A mesma situação foi provada com a resposta ao clopidogrel.^30^

Outro aspecto dos estudos sobre o paradoxo do fumante tem relação com desenho e metodologia. Quando observamos o desenho e os métodos estatísticos dos estudos alegando que existe um paradoxo do fumante, eles fornecem resultados hospitalares ou com acompanhamento relativamente curto, e a grande maioria utiliza o modelo de análise da regressão logística, que não considera o efeito de intervalos de tempo em relação ao evento e não lida com covariáveis tempo-dependentes. Encontramos somente um estudo que utilizou a análise de regressão de Cox e trouxe desfechos favoráveis para o ato de fumar^31^ por outro lado, a maioria dos estudos que encontrou desfechos desfavoráveis para o ato de fumar também utilizou a análise de regressão de Cox. Finalmente, nenhum dos estudos teve o objetivo de avaliar o paradoxo do fumante de forma primária. Este desenho sempre tem o risco de não detectar potenciais fatores de confusão.

A limitação mais importante deste estudo é o desenho retrospectivo. A aderência ao tratamento médico não foi conhecida após a alta. Não sabíamos se os pacientes continuavam a fumar após o STEMI, então, não foi possível chegar a uma conclusão sobre o efeito do fumo contínuo na mortalidade. Não temos informações sobre os desfechos cardiovasculares, como IM recorrente ou hospitalização por insuficiência cardíaca.

## Conclusão

O ato de fumar gradualmente aumenta o risco de mortalidade por todas as causas após o STEMI.
