# Supplementary material for: Efeitos do Ato de Fumar na Mortalidade de Longo Prazo após Infarto do Miocárdio por Elevação de ST
Source: Arq Bras Cardiol. 2021 Nov 17;118(1):24–32. [Article in Portuguese] doi: 10.36660/abc.20201036 (PMC8959056; doi:10.36660/abc.20201036)
Supplement: Translation

# Translation: Effects of Smoking on Very-Long Term Mortality after First ST Elevation Myocardial Infarction

**Authors:** Emrullah Kızıltunç, Yusuf Bozkurt Şahin, Salih Topal, Mehmet Akif Düzenli, Ekrem Karakaya, Nazif Aygül, Ramazan Topsakal, Kurtuluş Özdemir, Adnan Abacı

**Affiliations:** 1 Turkey Gazi University, School of Medicine, Cardiology Department, Ankara - Turkey; 2 Turkey Necmettin Erbakan University, School of Medicine, Cardiology Department, Konya - Turkey; 3 Turkey Karabük University, School of Medicine, Cardiology Department, Karabük - Turkey; 4 Turkey Selçuk University, School of Medicine, Cardiology Department, Konya - Turkey; 5 Turkey Erciyes University, School of Medicine, Cardiology Department, Kayseri - Turkey; 6 Turkey KTU Karatay University, Medicana Hospital, Cardiology Department, Konya - Turkey

**Keywords:** Tobacco Use Disorder, Nicotine/adverse effects, ST Elevation Myocardial Infarction/complications, Risk Factors, Mortality

## Abstract

**Background:**

The smoking paradox has been a matter of debate for acute myocardial infarction patients for more than two decades. Although there is huge evidence claiming that is no real paradox, publications supporting better outcomes in post-MI smokers are still being released.

**Objective:**

To explore the effect of smoking on very long-term mortality after ST Elevation myocardial infarction (STEMI).

**Methods:**

This study included STEMI patients who were diagnosed between the years of 2004-2006 at three tertiary centers. Patients were categorized according to tobacco exposure (Group 1: non-smokers; Group 2: <20 package*years users, Group 3: 20-40 package*years users, Group 4: >40 package*years users). A Cox regression model was used to estimate the relative risks for very long-term mortality. P value <0.05 was considered as statistically significant.

**Results:**

There were 313 patients (201 smokers, 112 non-smokers) who were followed-up for a median period of 174 months. Smokers were younger (54±9 vs. 62±11, p: <0.001), and the presence of cardiometabolic risk factors were more prevalent in non-smokers. A univariate analysis of the impact of the smoking habit on mortality revealed a better survival curve in Group 2 than in Group 1. However, after adjustment for confounders, it was observed that smokers had a significantly increased risk of death. The relative risk became higher with increased exposure (Group 2 vs. Group 1; HR: 1.141; 95% CI: 0.599 to 2.171, Group 3 vs Group 1; HR: 2.130; 95% CI: 1.236 to 3.670, Group 4 vs Group 1; HR: 2.602; 95% CI: 1.461 to 4.634).

**Conclusion:**

Smoking gradually increases the risk of all-cause mortality after STEMI.

## Introduction

The causal association between smoking and atherosclerotic cardiovascular diseases, malignancies and parenchymal pulmonary diseases has been documented.^1^ Despite this clear association, the smoker’s paradox (a better outcome after some particular life threatening diseases in smokers versus non-smokers) has been a matter of debate for many years. Despite the increasing evidence supporting there is not a real smoker’s paradox, there are up-to-date publications claiming there is a smoker’s paradox for some kind of diseases or patient groups, like those with acute myocardial infarction, stroke, and patients undergoing transcatheter aortic valve implantation.^2 - 4^ Low platelet reactivity, enhanced antiplatelet activity with clopidogrel and possible activation of the preconditioning pathways with smoking-induced hypoxia are being suggested as the background of the smoker’s paradox.^5 , 6^ Additionally, it is suggested that some regional disparities can be found for smoker’s paradox.^7 , 8^

In this study, we aimed to explore the effect of smoking with a dose-dependent approach on very long-term mortality in the survivors of first STEMI.

## Methods

### Study patients

This retrospective study included the first ever STEMI patients who were followed-up in three state University hospitals in Turkey between 2004-2006 (Gazi University, School of Medicine, Cardiology Department, Ankara; Erciyes University, School of Medicine, Cardiology Department, Kayseri; Selçuk University, School of Medicine, Cardiology Department, Konya). The study was conducted according to the Declaration of Helsinki, and ethical approval was obtained from the Ethical Committee of Gazi University, School of Medicine, also the other participating departments allowed to perform the study. STEMI was defined as the following: presence of typical chest pain or equivalent symptom, presence of ST segment elevation ≥ 2 mm in at least two continuous leads, and/or increased CK-MB concentrations. Patients who had indication for urgent reperfusion were treated with thrombolytic therapy or primary percutaneous coronary intervention (PCI). A rescue PCI was performed in patients whose thrombolytic therapy failed. Patients who had their symptoms relieved and whose EKG was consistent with subacute STEMI at the presentation, as well as patients whose thrombolysis was successful, underwent coronary angiography within 24 hours after hospital admission. All of the patients received acetylsalicylic acid 100 - 300 mg, clopidogrel 75 mg and statin (atorvastatin, simvastatin, pravastatin or fluvastatin) at discharge. More than 90% of the patients were on beta blockers (metoprolol or carvedilol) and renin angiotensin blocker treatment at discharge, unless there was any contraindication or intolerance to these agents.

During the study period, all STEMI patients were consecutively recruited into a STEMI database. All of the patients were questioned about symptoms, major classical risk factors, coronary heart disease and past medical history. Physical examination findings, laboratory findings including complete blood count, creatinine levels, blood glucose levels, serum lipids, peak creatine kinase levels, and peak creatine kinase MB levels were recorded prospectively at index hospitalization. Echocardiographic and angiographic data were collected. For smoking information, patients were asked if they had ever smoked a cigarette; if so, the number of cigarettes per day and the duration of the smoking was recorded, so the dose of smoking exposure was calculated as package*years [(number of cigarettes per day/20)*years of smoking]. Patients were categorized according to the amount of cigarette exposure (Group 1: newer smokers, Group 2: <20 package*years users, Group 3: 20-40 package*years users, Group 4: >40 package*years users). The following patients were excluded from the survival analysis; those who were diagnosed with non-ST elevation myocardial infarction; patients who had any heart disease other than index STEMI (heart failure, significant valvular heart disease); the ones who had previous history of myocardial infarction or a previous coronary revascularization procedure; and patients who had a poor acoustic window to perform transthoracic echocardiography. Individuals whose smoking information were lacking from the index hospitalization (including cigarettes consumed per day and years smoked), whose survival information was unachievable and who died at index hospitalization were also excluded.

### Echocardiography

The echocardiographic examinations were performed in a 2-day average (25th and 75th percentiles of 1-3 days) after admission using a Vingmed CFM System Five (GE Medical. Horten, Norway), with a 2.5-MHz transducer or a 5000 model, Advanced Technology Laboratories Inc; (Bothell, WA) with a 2 to 4 MHz transducer, being recorded on digital media. Standard parasternal long- and short-axis, apical 4- and 2-chamber views were recorded in the left lateral position at rest. The left ventricle was analyzed according to the 16-segment model as proposed by the American Society of Echocardiography.^9^ Regional wall motion in each segment was graded visually, using a four-point scoring system: 1= normal, normal wall motion; 2= hypokinesia, marked reduction in endocardial motion; 3= akinesia, absence of inward wall motion; 4= dyskinesia, paradoxical wall motion away from the left ventricular lumen in systole. If more than two segments in the infarct zone or 4 or more in all 16 segments were not visualized, the study was considered inadequate and these patients were not included in the study. The left ventricular wall motion score index (WMSI) and left ventricular ejection fraction (LVEF) were used to evaluate the extent of left ventricular systolic dysfunction. WMSI was calculated by dividing the sum of the segmental scores by the number of visualized segments. The modified biplane Simpson method was used to measure LVEF. Severe left ventricular dysfunction was defined as LVEF <40%. All echocardiograms were analyzed by two experienced observers who were blinded to the clinical and angiographic data.

### Coronary angiography

Coronary angiography was performed by femoral access using the standard Judkins technique. Coronary artery stenosis was visually estimated by two independent observers while blinded to the identity and clinical information of the patients. The location of the culprit lesion was determined with coronary angiography. Single vessel disease was defined by diameter stenosis higher than 50% in only one coronary artery. Two and three-vessel disease is defined according to the same criteria. Left main disease was regarded as two-vessel disease.

### Survival information

In May, 2020, the survival data of the patients were collected by the electronic death notification system, retrospectively. In our country, all of the deaths have to be recorded in the governmental electronic death notification system with the personal identification number. So, this system provides robust data about the survival information and date of death. The follow-up duration was calculated by extracting the diagnosis date from the date of death in dead patients, and by extracting the diagnosis date from May 1st, 2020, in alive patients.

### Statistical Analysis

The SPSS 22.0 software for Windows was used for data analysis. For continuous variables, the distribution normality was tested using the Kolmogorov-Smirnov test. The results were presented as mean ± standard deviation (SD) for variables with normal distribution, and as median (interquartile range 25-75) for variables with abnormal distribution. Categorical variables were presented with numbers and percentage rates. For the comparison of the continuous variables between smokers and non-smokers, independent samples from the t-test or the Mann-Whitney U test were used where appropriate. Categorical variables were analyzed using the Chi-square test or the Fisher’s exact test. The log rank test was used to detect univariate effects of the particular study variables on mortality. The Kaplan-Meier survival estimates were calculated. The possible factors identified in univariate analyses were further included in the Cox regression analysis to determine the independent predictors of all-cause mortality. P value <0.05 was considered as statistically significant.

## Results

Three hundred and thirteen consecutive patients diagnosed with acute or subacute STEMI were enrolled in the study. Median follow-up duration was 14.5 years. One hundred and twelve (35.8%) patients had never smoked before the index event (Group 1). The exposure amount of the smokers at the index event was as following; 66 patients (21.1%) <20 package*years (Group 2); 94 patients (30.0%) 20-40 package*years (Group 3); 41 patients (13.1) >40 package*years (Group 4).

Baseline demographic features and laboratory parameters of the smokers and non-smokers are shown in Table 1 . Smokers were younger and more frequently male. Hypertension and diabetes were more prevalent in non-smokers, and family history for coronary artery disease were more prevalent in smokers. While hemoglobin levels at admission were higher among smokers than non-smokers, total cholesterol, low density cholesterol and blood glucose levels were higher at admission in non-smokers than smokers. Infarction site, receiving an urgent reperfusion treatment, peak creatinine kinase (CK) and CK-MB levels, infarct-related artery (IRA) and Gensini scores were similar among smokers and non-smokers ( Table 2 ). Mean ejection fraction (EF) was higher in smokers than non-smokers.

**Table 1:** – Baseline demographic and laboratory features of smokers and non-smokers

Variables	Non-Smokers (112)	Smokers (201)	p
Age (years)	62±11	54±9	<0.001
Gender (Female)	30(26.8)	16(8.0)	<0.001
Hypertension	52(46.4)	46(22.9)	<0.001
Diabetes	28(25.0)	18(9.0)	<0.001
Family History for CAD	17(15.2)	50(24.9)	0.045
Tobacco Exposure - Grade			
<20 pack-years	-	66(32.8)	
20-40 pack-years	-	94(46.8)	
>40 pack-years	-	41(20.4)	
Hemoglobin, g/dl	14.0±1.7	14.7±1.5	<0.001
WBC*10^3^	10.8±3.3	11.8±4.0	0.022
Creatinine, mg/dl	1.08±0.26	1.03±0.22	0.120
Total cholesterol, mg/dl	198±44	187±41	0.025
LDL, mg/dl	131±37	121±36	0.017
HDL, mg/dl	41±10	40±11	0.521
Triglyceride, mg/dl	111(68-161)	113(82-164)	0.296
Blood Glucose at admission, mg/dl	148±74	125±45	0.002

*CAD: coronary artery disease, HDL: high density lipoprotein, LDL: low density lipoprotein, WBC: white blood cell count Continuous variables were presented as mean±SD or median (IQR 25-75); categorical variables were presented as number (%).*

**Table 2:** – Clinical, angiographic, echocardiographic features of smokers and non-

Variables	Non-Smokers (112)	Smokers (201)	p
Anterior MI, n(%)	65(58.0)	112(55.7)	0.692
Thrombolysis + Primary PCI, n(%)	90(80.4)	154(76.6)	0.444
Peak CK, U/l	2065(1239-2955)	2170(1361-3396)	0.253
Peak CK-MB, U/l	189(122-286)	225(134-360)	0.149
Ejection Fraction,%	47±10	50±9	0.036
Wall Motion Score Index	1.59±0.36	1.57±0.34	0.584
IRA, n(%)			
LAD	65(58.0)	113(56.2)	0.782
CX	8(7.1)	19(9.5)	
RCA	39(34.8)	69(34.3)	
Gensini	39(24-55)	38(18-52)	0.213
EF <40%, n(%)	21(18.8)	31(15.4)	0.448
Death, n(%)	38(33.9)	70(34.8)	0.873

*CK: creatine kinase; CX: circumflex artery; EF: ejection fraction; IRA: infarct-related artery; LAD: left anterior descending artery; MI: myocardial infarction; RCA: right coronary artery. Continuous variables were presented as mean±SD or median (IQR 25-75); categorical variables were presented as number (%).*

During follow-up, death occurred in 108 (34.5%) patients; 38 (33.9%) deaths occurred among non-smokers; 70 (34.8) deaths occurred among smokers (p=0.873). Table 3 shows the study variables in alive and dead patients. In the dead group, patients were older, had lower hemoglobin levels at admission, lower EF and higher WMSI after the ischemic event. Being female, a heavy smoker and presenting EF<40% were also more prevalent factors in the dead group. Table 4 demonstrates the multivariate Cox regression analysis for all-cause mortality. Older age, smoking and having an EF<40% after the infarction were the independent predictors of very long-term mortality. The relative risk for mortality was increasing with a dose-dependent manner in smokers compared to non-smokers. (Group 2 vs. Group 1; HR:1.141; 95%CI: 0.599 to 2.171, Group 3 vs. Group 1; HR:2.130; 95%CI: 1.236 to 3.670, Group 4 vs. Group 1; HR: 2.602; 95%CI: 1.461 to 4.634). Survival curves of smokers and non-smokers are shown in Figure 1 . In Figure 1A, survival curves of smokers and over all non-smokers were presented. In Figure 1B, the unadjusted analysis revealed that Group 2 was exhibiting a better survival curve than Group 1. However, after adjusting for age (Figure 1C) and all other confounders (age, hypertension, diabetes, hemoglobin, infarct-related artery, infarction site, receiving urgent reperfusion treatment and presence of a depressed left ventricle EF) (Figure 1D), the survival curves exhibited the dose-dependent increased risk of smoking.

**Table 3:** – Clinical, demographic, angiographic, echocardiographic features of the alive and deceased patients

Variable (number)	Alive (205)	Dead (108)	p
Age, years	54±10	62±10	<0.001
Gender (Female)	23(11.2)	23(21.3)	0.017
Hypertension	55(26.8)	43(39.8)	0.019
Diabetes	28(13.7)	18(16.7)	0.475
Family History for CAD	45(22.0)	22(20.4)	0.746
Tobacco Exposure Grade			0.021
Non smoker	74(36.1)	38(35.2)	
<20 pack-years	50(24.4)	16(14.8)	
20-40 pack-years	62(30.2)	32(29.6)	
>40 pack-years	19(9.3)	22(20.4)	
Hemoglobin, g/dl	14.6±1.5	14.2±1.8	0.027
WBC*10^3^	11.4±3.6	11.7±4.2	0.471
Creatinine, mg/dl	1.04±0.21	1.07±0.27	0.216
Total cholesterol, mg/dl	194±44	185±40	0.096
LDL, mg/dl	126±37	121±36	0.336
HDL, mg/dl	41±10	41±13	0.683
Triglyceride, mg/dl	115(82-175)	106(73-146)	0.055
Admission Blood Glucose, mg/dl	132±63	137±49	0.566
Anterior MI	118(57.6)	59(54.6)	0.619
Thrombolysis + Primary PCI	163(79.5)	81(75)	0.360
Peak CK, U/l	2156(1308-2999)	2172(1368-3726)	0.269
Peak CK-MB, U/l	202(118-300)	232(141-379)	0.050
Ejection Fraction, %	50±9	47±10	0.016
Wall Motion Score Index	1.54±0.31	1.64±0.40	0.013
IRA			0.979
LAD	117(57.1)	61(56.5)	
CX	18(8.8)	9(8.3)	
RCA	70(34.1)	38(35.2)	
Gensini Score	38(19-52)	38(21-57)	0.396
EF <40%	25(12.2)	27(25.0)	0.004

*CAD: coronary artery disease; CK: creatine kinase; CX: circumflex artery; EF: ejection fraction; HDL: high density lipoprotein; IRA: infarct related artery; LAD: left anterior descending artery; LDL: low density lipoprotein; MI: myocardial infarction; RCA: right coronary artery; WBC: white blood cell count. Continuous variables were presented as mean±SD or median (IQR 25-75); categorical variables were presented as number (%).*

**Table 4:** – Multivariate Cox regression analysis for the very long-term mortality

	Exp(B)	95%CI for Exp(B)		p
		Lower	Upper	
Age	1,063	1,040	1,088	0,000
Gender (Female)	1,730	0,985	3,040	0,056
Diabetes Mellitus	1,264	0,740	2,158	0,391
Hypertension	1,184	0,765	1,832	0,448
Smoking				0,003
Group 2 vs. Group 1	1,141	,599	2,171	0,689
Group 3 vs. Group 1	2,130	1,236	3,670	0,006
Group 4 vs. Group 1	2,602	1,461	4,634	0,001
Hemoglobin	0,978	0,856	1,118	0,749
Infarction Wall (non-anterior vs anterior )	0,771	0,257	2,307	0,641
Urgent reperfusion (acute presentation vs subacute presentation)	0,978	0,622	1,538	0,924
IRA				0,825
RCA vs LAD	1,305	0,428	3,982	0,640
CX vs LAD	1,089	0,313	3,790	0,894
EF<40%	1,967	1,216	3,181	0,006

CX: circumflex artery; EF: ejection fraction; IRA: infarct-related artery; LAD: left anterior descending artery; RCA: right coronary artery. Group 1: never smokers, Group 2: <20 package*year smokers, Group 3: 20-40 package*year smokers, Group 4: >40 package*year smokers.

**Figure 1:** – Kaplan-Meier survival curves of smokers and non-smokers; Overall smokers and non-smokers (A); unadjusted curves of smoking groups (B); adjusted for age (C); and adjusted for age, hypertension, diabetes, hemoglobin, infarct-related artery, infarction site, receiving urgent reperfusion treatment and presence of a depressed left ventricle EF (D); Group 1: non-smokers, Group 2: <20 package*years smokers, Group 3: 20-40 package*years smokers, Group 4: >40 package*years smokers.

## Discussion

The main finding of this study is that among patients who survived after STEMI, those who smoked before the infarction presented higher all-cause mortality than those who had never smoked. The relative risk for mortality grows with the increasing cigarette exposure. The other independent predictors are age and presence of a post-MI depressed left ventricle systolic function.

Many epidemiological studies have demonstrated that the risk associated with smoking increases in a dose-dependent manner.^10 , 11^ Nonetheless, it has been suggested that smokers have a better prognosis in some clinical situations. In addition to acute MI patients, the presence of a smoker’s paradox has been defined in patients with ischemic stroke, resuscitated in-hospital cardiac arrest patients, and TAVI patients.^12 - 14^ The presence of smoker’s paradox in patients with acute MI is controversial. The first studies about the smoker’s paradox were published in the thrombolytic era. Some of those studies suggested that smoking was independently associated with favorable outcomes, and others revealed that smokers had favorable outcomes only in univariate analysis.^15 , 16^ The structure of the culprit lesion in smokers (a larger thrombus burden in smokers than in non-smokers) had been discussed as a potential explanation for the smoker’s paradox in acute MI patients. It was suggested that culprit thrombus in smokers have a tendency towards spontaneous lysis and/or response in thrombolytic therapy, which would be better in smokers compared to non-smokers.^17^

The results of the studies during primary PCI were also controversial. Some studies showed that smokers and non-smokers had similar mortality rates; some other studies suggested that smoking was associated with favorable outcomes.^18 , 19^ Also, there were studies showing that smokers present worse outcomes.^20^ For example, it was demonstrated that smoking was an independent predictor of lower in-hospital mortality in patients with acute MI, even after multiple analysis to control for potential confounders.^21^ Another study found that smokers present lower acute inflammatory response, better microvascular reperfusion and better 30-day mortality rates in the setting of acute MI.^3^ Clopidogrel responsiveness in smokers has been the most popular mechanism proposed for smoker’s paradox in the primary PCI era. Lower platelet reactivity was observed in smokers treated with clopidogrel in comparison to non-smokers treated with clopidogrel.^22 , 23^ It was suggested that the regional disparities for smoker’s paradox presented and this hypothesis were based on the possible genetic variability of the metabolism of clopidogrel in different races.^8^

Although there are many studies approaching the smoker’s paradox, there are plenty of well-designed analyses which demonstrated opposite findings. Five-year results of the SYNTAX trial demonstrated that smoking was associated with poor outcomes after revascularization in patients with complex coronary artery disease.^24^ The ACUITY trial established that among patients who were diagnosed with non-ST elevation MI, those who smoked presented a higher one-year mortality rate than those who did not smoke.^25^ The mortality rate in smokers was found to be significantly higher than in non-smokers in a large registry of STEMI patients who were treated with primary PCI.^26^ Additionally, cardiac MRI studies revealed that among STEMI patients, those who smoked had higher rates of myocardial hemorrhage and worse cardiovascular outcomes.^27^ Recent studies rejected the presence of smoker’s paradox in patients with heart failure and in patients with acute ischemic stroke.^28 , 29^ And finally, the result of the study published by Kim et al.^30^ was quite remarkable. They found that the difference of clopidogrel responsiveness in smokers was largely attributable to the difference in hemoglobin levels. They found similar platelet reactivity between smokers and non-smokers after adjusting the influence of hemoglobin on platelet reactivity.^30^

In our study, the unadjusted analysis revealed that mild smokers had a better survival curve than non-smokers. This can lead to the idea that mild smoking may be a good habit, but after adjustments according to confounding factors, smoking increased the risk of mortality in a dose-dependent manner. So, baseline confounding factors are very important for the smoker’s paradox. As we mentioned before, many of the studies revealed that the favorable outcomes attributed to smoking vanished after multivariate analysis. The same situation has also been proven for clopidogrel responsiveness.^30^

Another aspect of smoker’s paradox studies regards design and methodology. When we look at the design and the statistical methods of the studies claiming there is a smoker’s paradox, they provide in-hospital or relatively short-term follow-up results, and the vast majority used the logistic regression analysis, which does not consider the effect of time intervals to the event and cannot handle time-dependent covariates. We could find only one study which used the Cox regression analysis and found favorable outcomes for smoking;^31^ on the other hand, most studies that found unfavorable outcomes for smoking used the Cox regression analysis. And finally, none of the studies were designed to evaluate the smoker’s paradox primarily. This design has always the risk of undetected potential confounders.

The most important limitation of this study is the retrospective design. Adherence to the medical treatment was not known after discharge. We did not know which patients continued to smoke after the STEMI, so we could not draw a conclusion on the effect of ongoing smoking on mortality. We did not have the information about cardiovascular endpoints, like recurrent MI or hospitalization for heart failure.

## Conclusion

Smoking gradually increases the risk of all-cause mortality with after STEMI.
